# Brief Notes

**Published:** 1914-03

**Authors:** 


### BRIEF NOTES

Bed Bugs. Hoosain of India recommends equal parts of spirits of turpentine and soap suds, the latter preventing too rapid evaporation of the former and making it stick to the smooth and hard backs of the bugs.

Salvarsan or Neosalvarsan has been used successfully in pernicious anæmia—but allowance should be made for the frequent long remissions of this disease.

Absolute (?) Test of Death. Icard of Marseilles advises injecting a solution of fluorescin. If there is the slightest blood current, the body becomes a vivid golden yellow, the eyes a deep emerald green. Half an hour is long enough for the test. In death, there being no circulation, the dye is not diffused. The test is harmless if life is present, as the dye is quite rapidly eliminated.

Bismuth Oxid is advised by Lion of Paris for X-ray work, on account of forming a harmless, insoluble oxy-chlorid with gastric juice.

Membranous Interitis. H. Roger of Paris claims that the intestine secretes a mucus-coagulating ferment, mucinase, but that this is normally inhibited by bile. Hence, he advocates the administration of bile when there are tough membranes passed. (But, hydrobilirubin is not regularly lacking in such cases, and it is often deficient when membranes are not present. Besides, we are somewhat skeptic as to the facility with which ferments are being discovered.—Ed.)

Repeated Extra-Uterine Pregnancy. S. Higuchi of Japan reports (*Sei-I-Kwai,* December, 1913) cases occurring in the same woman in 1906 and 1913, with recovery.

Disinfection of Typhoid Stools. Cover with water at 50-60 degrees C. (122-140 F.) Add a cupful of quick lime, let stand 1½ hours. The U. S. P. H. S. declares that this developes enough heat to sterilize completely.

